# Haematological and Biochemical Reference Values for Healthy Population of Maferinyah Rural Community in Guinea

**DOI:** 10.1155/2020/8605485

**Published:** 2020-08-03

**Authors:** Abdoul Habib Béavogui, Almamy Amara Touré, Daouda Camara, Abdoulaye Doumbouya, Malick Minkael Sylla, Sékou Touré, Ahmadou Hamidou Togo, Mamadou Saliou Diallo, Alexandre Delamou, Issaka Sagara, Alassane Dicko, Abdoulaye Djimdé

**Affiliations:** ^1^Centre National de Formation et de Recherche en Santé Rurale de Maferinyah, Forecariah, Guinea; ^2^Bioclinical and Fundamental Sciences Chair, Department of Medical Sciences, Faculty of Health Science and Techniques, Gamal Abdel Nasser University of Conakry, Conakry, Guinea; ^3^Malaria Research and Training Center (MRTC), Département d'Epidémiologie des Affections Parasitaires (DEAP), Faculté de Médecine de Pharmacie et d'Odonto-Stomatologie (FMPOS), Université des Sciences, des Techniques et des Technologies de Bamako (USTTB), Mali; ^4^Department of Public Health, Faculty of Health Sciences and Techniques, Gamal Abdel Nasser University, Conakry, Guinea; ^5^Centre d´Excellence Africain pour la Prévention et le Contrôle des Maladies Transmissibles (CEA-PCMT), Gamal Abdel Nasser University, Conakry, Guinea

## Abstract

Guinea's reference ranges for biological parameters rely on those of Caucasian values. Variability in reference ranges according to the context is well-documented. We conducted this study for the purpose of future malaria clinical trials that assess the efficacy and safety of malaria drugs. A repeated cross-sectional study was carried out, in an apparently healthy cohort population. Surveys took place in Maferinyah rural community, which is located at 75 km from the capital. The 2.5^th^ and 97.5^th^ percentiles were determined nonparametrically and stood for reference intervals. Reference values were determined separately for males and females according to ranges of age (6-10 years of age; 11-15 years of age; 16-45 years of age). Differences between genders were tested using the Mann-Whitney test, while the Friedman test was performed to test differences within each gender group according to the seasons. A total of 450 volunteers were enrolled. The median age was 13. Males 16-45 years of age had significantly higher hematologic and biochemical values compared to a female of the same age (for hematological parameters: Mean Cell Hemoglobin Concentration MCHC *p* ≤ 0.001, Platelets *p* ≤ 0.001, monocytes *p* = 0.0305, eosinophils *p* = 0.0225; for biochemical parameters: Aspartate aminotransferase AST *p* ≤ 0.001, Alanine Aminotransferase ALT *p* ≤ 0.001, creatinine *p* ≤ 0.001). We noticed significant seasonal variations for all the biochemical parameters and some hematologic parameters (Mean Corpuscular Hemoglobin MCH, MCHC, Mean Cell volume). This is the first study establishing hematologic and biochemical parameters in Guinea. These findings provide a useful guide for the clinical researchers and care providers. Studies on large scale and in different settings would be also desirable.

## 1. Introduction

Patient management relies heavily on the speed of clinical laboratory results, upon which medical diagnosis is established [1, 2]. Health care professional needs to draw a conclusion based on laboratory tests results, which depends on preanalytical and analytical factors [3–5]. These factors may influence laboratory results and may have an impact on hematological and biochemical tests' results. For instance, physiological factors that may influence laboratory results include age, activity, bed rest, food ingestion, alcohol ingestion, menstrual cycle, obesity, oral contraceptives, posture, pregnancy, race, gender, smoking, and time of day [4]. Variations of reference values due to physiological and demographical factors such as gender, age, regions, are well-documented [6–12]. Hence, the provision of reliable reference intervals remains an important task for clinical laboratories and diagnostic test manufacturers [13]. As a result, an expert committee in the US has recommended that each manufacturer of diagnostic kit must provide reference intervals [14]. Laboratories have also been instructed to validate the reference ranges and prove that they are applicable for their environment and populations [14].

In most developing countries, including Guinea, laboratory reference values in use are from Caucasian data. However, these developing countries do not have the same physiological and environmental patterns as industrialized countries. Their health conditions and the distribution of healthy populations are totally different from that of developed countries. The Mafèrinyah training and research center in Guinea is planning to conduct a phase-III clinical trial to assess antimalarial drug safety. This requires the use of local population reference values for Alanine aminotransferase (ALAT), Aspartate aminotransferase (ASAT), bilirubin, creatinine, and blood count in order to follow biological safety of these new agents. Unfortunately, there were no such reference values to define participants' enrollment criteria. Our study aimed at establishing reference values of haematological and biochemical parameters in healthy population in Mafèrinyah subprefecture.

## 2. Methodology

### 2.1. Study Design, Site, and Population

This was a cross-sectional study with a cohort participant. Three surveys were carried out between March 2011 and December 2013; the first one in the middle of the dry season (March), the second one at the start of the rainy season (May), and the last one at the end of the rainy season (November). Subprefecture of Mafèrinyah is part of the Prefecture of Forécariah. The population of the subprefecture of Mafèrinyah was estimated at 38,934 inhabitants and distributed between 6 villages as follows: Mafèrinyah centre: 14,595 inhabitants; Madinagbé: 8,321 inhabitants; Moribayah: 4,951 inhabitants; Fandié: 3,636 inhabitants; Koket: 3,023 inhabitants; and Maléah: 2,086 inhabitants. Mafèrinyah centre, which hosts the health Centre (CS) and the Centre for Training and Research in Rural Health (CFRSR), is located at 75 km from the capital Conakry and halfway (25 km) between the prefectures of Coyah and Forécariah (Unpublished data: Monitoring of health centre activities 2006). Participants in this study were from the Maferinyah community and aged 6-45 years old. A total of 450 volunteers were enrolled. Inclusion criteria included those who lived in the area for at least 6 months, nonpregnant healthy women, apparently healthy clinically, willingness to participate to the study without a long-term travel. Patients with confirmed malaria and those who do not want to participate to the study were excluded. The sample size was computed according to the Clinical and Laboratory Standards Institute (CLSI) guidelines for the establishment of reference interval [13], which recommends a minimum of 120 participants in each category used for stratification, we therefore completed this size to 150 participants in each category to take into account the loss of participants.

### 2.2. Survey Collection Procedure

Data were collected at Mafèrinyah Centre using a standardized individual questionnaire developed according to the objectives of the study. All participants meeting the inclusion criteria were enrolled. Data on general information, complete clinical examination, and biological examination were performed. Malaria diagnostic was performed for all participants. That information was transcribed into the case report forms by study clinical investigators. Clinical examination was carried out by the physician of the research team in order to make sure that all participants met the inclusion criteria.

### 2.3. Ethical Considerations

Ethical approval was obtained from the National Ethics Committee of Guinea called “*Comité National de l'Ethique de la Recherche en Santé de Guinée*” (Protocol number: 07/CNERS/10). Written informed consent was obtained from all participants after the purpose of the study was fully explained. For participants under 18, parents or guardians signed for them. For participants unable to read the French language, the content of the informed consent form was translated into the local language in the presence of an independent witness.

### 2.4. Laboratory Analysis

#### 2.4.1. Blood Collection

About 5 ml of venous blood were collected in an ethylene diamine tetra acetic acid (EDTA) vacutainer tube for haematology and in a serum separator vacutainer tube for biochemistry. All samples were kept at laboratory temperature and away from heat and sunlight.

#### 2.4.2. Hematologic Analysis

Haematological analyses were performed by using validated ABX Pentra 60 Hematology Analyzers (Horiba-ABX, Montpellier, France). Reagents, calibrators, and controls were provided by the manufacturer from the kit. Controls were run daily and calibrators each time a new batch of controls arrived. Samples analyses were performed within 4 hours after blood collection, and the results were printed for each participant and kept in his/her case report form (CRF).

#### 2.4.3. Biochemical Analysis

Biochemical analyses were performed by using Piccolo® xpress™ Chemistry Analyzer (USA). Test tubes, Reagents, calibrators, and controls were provided by Elitech Diagnostics (Sees, France). Blood samples for biochemical analyses were centrifuged, and serum was collected and analyzed within 4 hours, and the results were printed for each participant and kept in his/her CRF.

#### 2.4.4. Pregnancy Test

A pregnancy test was offered to female participants who were at least 13 years old prior to their enrollment. We used the method of urine strip, and all positive cases were excluded.

#### 2.4.5. Quality Control

Controls were run on the instrument daily according to the manufacturer recommendations. No analysis was performed if any control was out of range. In addition, an internal monitoring system was implemented to assess all the laboratory processes. Moreover, Mafèrinyah's research laboratory was subjected to external quality assessments for haematology and biochemistry by the Malaria Research and Training Centre of Bamako (Mali). The laboratory also complied with the principles of Good Clinical and Laboratory Practice (GCP/GLP) [15, 16].

### 2.5. Data Management and Statistical Analysis

Data were double-entered into an Access database and checked visually. The 2.5^th^ and 97.5^th^ percentiles were determined nonparametrically and stood for reference intervals [13]. Outliers within each subgroup were identified using the Dixon method [17]. To sum up, the extreme values were retained in the distribution if D/R, 0.33, where *D* is the absolute difference between the most extreme distribution and the next value and *R* is the Range (maximum–minimum). Reference values were determined separately for males and females according to the range of age (6-10 years of age; 11-15 years of age; 16-45 years of age). Differences between genders were tested using the Mann-Whitney test, while the Friedman test was performed to test differences within each gender group according to seasons. For the multiple comparison of groups that show the difference in variances Nemenyi was used. Data analysis was carried out using R 3.6.2. All tests were considered significant with *p* < 0.05.

## 3. Results

A total of 450 participants 6-45 years of age were enrolled. The median age was 13, the majority of participants were students (71%); 53% of study population were females; the ethnic group Soussou accounted for 65% of study sample (additional file). In participants of 6-10 years of age, males had significantly higher White Blood Cell (WBC), eosinophils, Aspartate Aminotransferase (AST), and total bilirubin (T/Bil) values compared to females (Table 1). However, Mean Cell Volume (MCV), lymphocytes, and basophils values were significantly higher in female participants than in males (Table 1). In the subgroup of 11-15 years of age, males had significantly higher hemoglobin, hematocrit, eosinophils, basophils, AST, ALT, T/Bil, and creatinine than in female (Table 2), female MCV, Mean Corpuscular Hemoglobin (MCH) values were significantly higher than in males (Table 2). In the subgroup 16-45 years of age, males had significantly higher values of red blood cells, hemoglobin, hematocrit, Mean Cell Hemoglobin Concentration (MCHC), Platelets, monocytes, eosinophils, AST, ALT (Alanine aminotransferase), and creatinine compared to female (Table 3); but at the same time, WBC, MPV, and neutrophils were significantly higher in female participants compared to those in male (Table 3). Reference values of females 6-10 years of age varied significantly between three (3) rounds for the following parameters: hemoglobin, hematocrit, MCV, AST, ALT, TBIL, and creatinine (Table 4). In a pairwise analysis, the following parameters, hematocrit, T/Bil, and creatinine, showed a significant difference between the first and the second round on one hand and the first and the third round on other hand (additional file). Conversely, ALT yielded a significant difference between the 1st and third round on one hand and between 2nd and third round on the other hand. MCV only shows a significant change between March and November while AST shows a significant difference between all the rounds (additional file). Reference values of females 11-15 years of age varied significantly between the three rounds for the following parameters: WBC, hematocrit, MCH, MCHC, MPV, AST, monocytes, AST, ALT, and T/Bil (Table 5). Pairwise analysis indicated significant WBC and MCH variation between the 1st and the 2nd round, between 2nd and the 3^rd^ round; we noticed the same result for T/Bil (additional file). Conversely, Hematocrit varied significantly between March and May on one side, and between March and November on the other side; MPV changed significantly between March and November (additional file). MCHC, AST, and ALT varied significantly between all rounds (additional file). Reference values of females from 16 to 45 years varied significantly between the three rounds for the following parameters: CCMH, AST, ALT, and TBILIRUBINE (Table 6). We observed significant changes in MCHC value between March and May and between March and November, but no significant variation between May and November (additional file). ALT and AST varied significantly between all rounds whereas T/Bil varied only between March and May and between March and November (additional file).

Reference values of males 6-10 years of age varied significantly between the three rounds for the following parameters: RBC, Hematocrit, MCV, MCH, MCHC, Neutrophils, Basophils, AST, ALT, T/Bil, and Creatinine (Table 7). In pairwise analysis, RBC, MCH, and neutrophils varied significantly between March and November. Hematocrit value changed significantly between March and May, and between March and November. We noticed significant variation in MCV values between March and May, and between May and November. CMCH, basophils/Bil, and creatinine changed significantly between March and May, and between March and November. ALT values changed significantly between March and November, and between May and November. We also observed significant variation in AST values between all seasons. Reference values of males 11-15 years of age varied significantly between the three rounds for the following parameters: Hematocrit, MCH, MCHC, AST, ALT, and T/Bil (Table 8). In addition, Hematocrit, MCHC, and T/Bil show significant variation between March and May, and between March and November; MCH varied significantly between May and November. AST and ALT values changed significantly between seasons. Reference values of males from 16 to 45 years of age varied significantly between the three rounds for the following parameters: Hematocrit, MCH, MCHC, MPV, monocytes, Neutrophils, AST, ALT, and T/Bil (Table 9). We noticed significant variation in hematocrit, MCH, and MCHC between March and May, and between March and November. MPV ad neutrophils values only vary significantly between March and November. Conversely, monocytes change significantly between March and May, and between May and November. We observed significant variation in ALT and T/Bil values between March and November, and between May and November. AST changed significantly between all rounds.

## 4. Discussion

Establishing the ranges of value is a key step toward clinical trials. Clinical and routine laboratories use Caucasian values to make decision in Guinea. However, socioeconomic and environmental (seasons) conditions may influence hematologic and biochemical parameters. It appears the need for having its own values as recommended by the Experts [14]. To the best of our knowledge, this is the first study ever done in Guinea, in order to address the malaria clinical trials' need, which must rely on context-based values. Hence, take into seasonal variations are more realistic since malaria is seasonal dependent. Our study highlighted an important variability in hematologic and biochemical values that will serve as well as routine and clinical laboratories.

On the overall rounds (*R*1 + *R*2 + *R*3), males had the highest levels of hemoglobin, hematocrit, basophils, AST, ALT, T/Bil (total bilirubin), and creatinine. Males have usually higher hematologic and biochemical counts than female, because of hormonal influence like androgen [18, 19]; according to Daniel the increase in size and volume of muscle fibers is an associated increase in the number of red blood cells, and, therefore rise in hematocrit and hemoglobin values [19]. In addition to that, menstrual period may lead down women RBC count. Our findings comply with several studies [10, 12, 20–24]. Conversely, females had higher levels of MCV and MCH and involved those of 6-15 years of age. Similarly, we hypothesize that WBC elevation in female adults compared to male ones is due to multiples aggressions (infectious diseases, injuries) that men are exposed in that context. Those findings are in accordance with the previous reports [9, 25]. We also found a decrease of WBC levels as age increases, which is in line with previous studies [7, 26]. In brief, we noticed few differences between females and males of 6-10 years of age, in fact, only 7 out of 18 parameters were significant. However, variations are more remarkable in males and females 11-15 years of age and 16-45 years of age with, respectively, 11 out of 18 parameters and 14 out of 18 parameters significant. This suggests that hematologic parameters change a little in early life regardless of age and gender [27]. We noticed few changes in female 6-10 years of age parameters, only 6 out of 18 parameters varied over time. Hematologic and biochemical parameters remained higher at the dry season compared to rainy seasons. The study period might explain that result, in fact, the two last surveys took place after 2 years of the first one, because of logistical reasons. Another relevant reason is the possible impact of malaria, even participants were careful to screen, and there might be some subclinical and submicroscopic cases. As females grew up the parameters change significantly, among 11-15 years of age cohort, 10 out 18 parameters had varied significantly through the rounds. We have already discussed about the influence of the age on parameter levels [7]. Females experienced various modifications such as the hormonal effect that lead to the menstrual period. Hematocrit, ALT, AST, and T/Bil decreased over time while MCH, MCHC, and MPV increased over time. The WBC and monocytes fluctuate through all the seasons. Interestingly, we found few parameter changes in female cohort adult, only 4 out of 18 parameters varied over time. MCH fluctuated trough the seasons, whereas AST, ALT, and T/Bil have decreased over time. On the contrary to females of the same age, most of the 6-10 years of age males' parameters changed through seasons. So, 12 out of 18 parameters varied according to seasons. Hematocrit, basophils, AST, ALT, T/Bil, and creatinine decreased significantly between dry season and end of the rainy season, while neutrophils, MCH, and MCHC increased significantly over time. As we were working on different times of day, this may change some parameters count. Diurnal variation of the neutrophils count has been established with the highest levels in the afternoon and lowest levels in the morning at rest [1]. In addition, as we have previously seen before, AST remained the only parameter that changed between all rounds.

Males of 11-15 years of age experienced few hematologic and biochemical parameters changes compared to females of the same age. We found 7 out of 18 parameters that varied through the periods. As we have seen before, Hematocrit and Biochemicals parameters (AST, ALT, and T/Bil) decreased significantly between the three rounds while MCH, MCHC increased significantly over time. AST and ALT values changed significantly through all the seasons. Many studies pointed out the impact of seasons on biochemical and haemotogical parameters; these include environmental factors (temperature, exposure to sunlight, climate, etc.), individual's factors (exercise, physical activity, diet, etc.) [28–34]. Of note that is, the rainy season is the favorable time in diseases increase like malaria and other parasitic diseases not screen in this study. Besides, other characteristics not studied in this study might explain the seasonal variations. For instance, one could seek to know if the harvest period influences the nutritional state and subsequently the parameter values. Therefore, specific study design to seasonal variations would be interesting.

Cohort adult males 16-45 years of age had slightly different changes in hematologic and biochemical parameters compared to females of the same age. We noticed 9 out of 18 parameters significantly varied over time. So, hematocrit, MPV, monocytes, AST, ALT, T/Bil, and Creatinine decreased significantly between seasons while MCH, MCHC, and Neutrophils raised over time. We failed to know if participants were nonsmokers or smokers, because we were only relying on participants' statements. It has been reported that cigarette smokers have higher average leukocyte counts than nonsmokers [1]. The increase is greatest (about 30%) in heavy smokers who inhale and affects neutrophils, lymphocytes, and monocytes [1]. We also notice again AST change between all rounds. Our study has limitations and strengths, we did not perform all the analysis that are needed to rule out diseases among volunteers like HIV, hepatitis C, syphilis, sickle cell disease, thalassemia, and micronutrient deficiencies. To fill this gap, we performed careful clinical evaluations through well-trained physicians. Only some parameters that we needed for malaria clinical trial assessment were included. Stratification in ranges of age according to gender has also affected the sample size. We consider that other studies on hematologic and biochemical parameters that will include more parameters, and diverse participants from different regions will reinforce evidences. In the meantime, these findings remain useful in the scarcity of data. Hence, we recommend its use by clinical laboratories.

## 5. Conclusion

Our study reveals interesting patterns of hematologic and biochemical parameters in Guinea. Little change in parameters was observed in males and females aged 6-15 years old. However, in accordance with previous reports, males' adults had higher parameters than females. We noticed parameter variations through the seasons mostly for few hematologic parameters (MCH, MCHC, and MCV) and for all the biochemical tests (ALT, AST, creatinine, and T/Bil). AST remained the only parameter that has changed through all the seasons. These results provide a useful guide for the clinical researchers and care providers.

## Figures and Tables

**Table 1:** Hematological and biochemical reference values of participants, for 6-10 years of age (*R*1 + *R*2 + *R*3)^**£**^.

Parameters	Female			Male			*p* value”
	*N*	Median	Reference values^∗^	*N*	Median	Reference values	
WBC (1000/mm^3^)	208	7.00	4.30-12.92	235	7.60	4.59-14.35	0.0278
RBC (10^6mm^3^)	208	4.49	3.53-5.38	235	4.48	3.36-5.46	0.8788
Hemoglobin (g/dl)	208	11.10	8.59-13.00	235	11.10	8.09-12.72	0.0852
Hematocrit (%)	208	35.10	25.789-41.00	235	34.60	25.87-39.55	0.2309
MCV (*μ*mm^3^)	208	78.00	62.85-89.00	235	77.00	62.70-88.00	0.0428
MCH (pg)	208	25.20	19.64-28.88	235	24.80	18.81-28.20	0.4986
MCHC (g/dl)	208	32.10	28.90-34.70	235	32.30	28.79-34.81	0.2688
Platelet (1000/mm^3^)	208	289.00	91.60-457.15	235	301.00	39.85-458.70	0.2688
MPV (*μ*m^3^)	208	7.90	6.70-10.06	235	8.10	6.50-9.73	0.8566
Lymphocytes (%)	208	50.30	31.41-63.43	235	45.90	28.47-61.45	≤0.001
Monocytes (%)	208	9.90	6.623-16.78	235	10.20	6.79-18.02	0.2581
Neutrophils (%)	208	34.10	20.20-52.95	235	35.90	21.44-57.65	0.1342
Eosinophils (%)	208	3.20	1.10-15.55	235	4.40	1.20-25.26	≤0.001
Basophils (%)	208	0.80	0.40-2.62	235	0.70	0.40-1.81	≤0.001
AST (UI/L)	208	29.80	12.99-51.24	235	32.00	14.65-59.32	0.0057
ALT (UI/L)	208	19.00	9.10-37.63	235	19.45	9.10-54.65	0.3069
T/Bil (mg/L)	208	0.50	0.20-1.42	235	0.60	0.20-2.12	0.0074
Creatinine (mg/L)	208	0.54	0.24-0.73	235	0.53	0.30-0.81	0.7588

^**£**^ *R*1: first round, *R*2: second round, *R*3: third round. ^∗^References values stand for 2.5^th^-97.5^th^ percentiles. WBC: white blood cell, RBC: red blood cell, MCV: mean cell volume, MCH: mean corpuscular hemoglobin, MCHC: mean cell hemoglobin concentration, MPV: mean platelet volume, ALT: alanine aminotransferase, AST: aspartate aminotransferase, T/Bil: total bilirubin. ”*p* value based on Mann-Whitney test.

**Table 2:** Hematological and biochemical reference values of participants, 11-15 years of age (*R*1 + *R*2 + *R*3)^£^.

Parameters	Female			Male			^∗^ *p* value
	*N*	Median	Reference values	*N*	Median	Reference values	
WBC (1000/mm^3^)	209	6.60	3.8-11.5	150	6.40	4.09-10.80	0.5199
RBC (10^6mm^3^)	209	4.56	3.82-5.49	150	4.57	3.75-5.81	0.9868
Hemoglobin (g/dl)	209	11.80	9.50-13.73	150	11.50	9.00-14.05	0.0027
Hematocrit (%)	209	37.20	28.64-43.80	150	36.20	29.34-45.28	0.0086
MCV (*μ*mm^3^)	209	82.00	67.85-93.00	150	81.00	65.85-90.00	0.0012
MCH (pg)	209	26.00	20.07-30.90	150	25.30	19.57-29.10	0.0141
MCHC (g/dl)	209	31.70	29.085-35.20	150	31.30	28.89-34.70	0.2229
Platelet (1000/mm^3^)	209	272.00	94.70-441.65	150	260.00	110.25-409.25	0.3470
MPV (*μ*m^3^)	209	8.30	6.90-10.03	150	8.10	6.67-10.00	0.0068
Lymphocytes (%)	209	46.50	29.51-61.72	150	44.70	30.495-59.10	0.1745
Monocytes (%)	209	10.50	6.470-16.21	150	10.20	6.34-18.10	0.9745
Neutrophils (%)	209	36.20	20.70-56.11	150	35.40	19.89-52.82	0.1544
Eosinophils (%)	209	3.60	1.10-20.25	150	6.10	1.49-18.62	≤0.001
Basophils (%)	209	0.60	0.40-1.00	150	0.70	0.39-5.41	0.0015
AST (UI/L)	209	27.00	10.66-40.00	150	31.30	13.31-52.00	≤0.001
ALT (UI/L)	209	17.00	9.10-28.30	150	19.00	9.10-40.747	0.0202
T/Bil (mg/L)	209	0.50	0.20-1.12	150	0.50	0.20-1.32	≤0.001
Creatinine (mg/L)	209	0.62	0.29-0.92	150	0.70	0.39-1.00	0.0046

^∗^ *p* value based on Mann-Whitney test. ^**£**^(*R*1: first round; *R*2: second round; *R*3: third round).

**Table 3:** Hematological and biochemical reference values of participants, 16-45 years of age (*R*1 + *R*2 + *R*3) ^£^.

Parameters	Female			Male			^∗^ *p* value
	*N*	Median	Reference values	*N*	Median	Reference values	
WBC (1000/mm^3^)	206	6.40	4.00-10.43	208	6.00	3.90-10.62	0.0408
RBC (10^6mm^3^)	206	4.41	3.63-5.71	208	5.04	4.23-6.10	≤0.001
Hemoglobin (g/dl)	206	11.60	8.83-13.60	208	13.40	8.20-15.50	≤0.001
Hematocrit (%)	206	36.70	28.64-43.80	208	41.90	29.74-48.16	≤0.001
MCV (*μ*mm^3^)	206	83.00	63.85-97.00	208	83.00	62.70-95.15	0.7061
MCH (pg)	206	26.40	19.35-31.22	208	26.80	18.50-31.35	0.1172
MCHC (g/dl)	206	26.50	28.38-34.40	208	32.30	29.04-34.82	≤0.001
Platelet (1000/mm^3^)	206	201.20	86.1-413.6	208	226.00	54.95-526.15	≤0.001
MPV (*μ*m^3^)	206	8.40	7.00-10.12	208	8.30	6.80-10.10	≤0.001
Lymphocytes (%)	206	43.90	27.30-62.28	208	45.00	26.00-63.40	0.2118
Monocytes (%)	206	9.30	5.40-15.56	208	9.90	6.99-17.29	0.0305
Neutrophils (%)	206	39.60	22.71-60.93	208	35.50	20.57-60.03	0.0088
Eosinophils (%)	206	3.90	0.99-20.04	208	5.20	1.10-22.64	0.0225
Basophils (%)	206	0.60	0.39-1.43	208	0.60	0.40-1.30	0.0068
AST (UI/L)	206	25.00	10.37-44.69	208	28.60	13.56-49.15	≤0.001
ALT (UI/L)	206	18.00	9.10-47.14	208	20.00	9.10-46.09	0.0042
T/Bil (mg/L)	206	0.50	0.20-1.20	208	0.60	0.20-2.22	0.7418
Creatinine (mg/L)	206	0.76	0.30-1.03	208	0.97	0.29-1.31	≤0.001

∗*p* value based on Mann-Whitney test. ^**£**^(*R*1: first round; *R*2: second round; *R*3: third round).

**Table 4:** Reference values among Female cohort 6-10 years of age who attended all rounds (1 to 3). *N* = 49.

Parameters (unit)	1st round (dry season)		2nd round (rainy season)		3rd round (end of rainy season)		^∗^ *p* value
	Median	References value	Median	References value	Median	References value	
WBC (1000/mm^3^)	8.10	4.50-16.23	7.60	4.55-12.80	7.60	4.85-12.32	0.2596
RBC (10^6mm^3^)	4.56	3.44-5.71	4.50	3.88-5.37	4.33	3.25-5.20	0.0797
Hemoglobin (g/dl)	11.20	7.83-12.19	11.10	9.60-12.70	11.10	8.04-13.15	0.0137
Hematocrit (%)	36.80	26.62-40.34	33.10	28.60-37.70	33.90	25.65-39.35	0.0011
MCV (*μ*mm^3^)	78.00	15.88-88.00	74.00	59.50-85.50	79.00	67-85	0.0946
MCH (pg)	23.75	19.81-27.34	24.80	19.55-28.70	25.80	21.23-28.21	0.0074
MCHC (g/dl)	30.40	28.71-31.95	33.40	31.50-35.10	32.70	30.50-34.77	≤0.001
Platelet (1000/mm^3^)	305.00	60.88-445.62	288.00	88.20-467.50	304.00	151.25-476.75	0.5523
MPV (*μ*m^3^)	8.20	6.91-9.29	8.20	6.80-9.80	7.90	6.8-9.7	0.0196
Lymphocytes (%)	47.60	28.94-61.96	46.40	27.25-60.15	43.70	31.06-62.38	0.1301
Monocytes (%)	10.45	6.83-15.64	9.80	6.95-15.30	10.50	6.48-19.09	0.1919
Neutrophils (%)	34.10	21.66-53.11	35.90	22.20-58.95	36.30	21.67-67.38	0.348
Eosinophils (%)	4.40	0.85-20.76	4.70	1.20-20.70	4.20	1.03-26.76	0.4444
Basophils (%)	0.75	0.41-3.64	0.60	0.35-0.90	0.70	0.45-1.05	0.0761
AST (UI/L)	37.00	29.00-55.75	31.70	21.15-57.45	23.10	13.08-57.24	≤0.001
ALT (UI/L)	22.00	16.12-50.25	20.90	10.81-59.84	10.65	9.10-35.85	≤0.001
T/Bil (mg/L)	0.60	0.40-2.54	0.30	0.20-0.85	0.40	0.20-1.17	≤0.001
Creatinine (mg/L)	0.50	0.30-0.70	0.58	0.40-0.69	0.56	0.39-0.77	0.6065

^∗^ *p* value based on Friedman test.

**Table 5:** Reference values among Female cohort 11-15 years of age who attended all rounds (1 to 3). *N* = 40.

Parameters (unit)	1st round (dry season)		2nd round (rainy season)		3rd round (end of rainy season)		^∗^ *p* value
	Median	References value	Median	References value	Median	References value	
WBC (1000/mm^3^)	6.60	4.19-8.56	7.30	4.9-11.9	5.85	4.30-12.52	0.0019
RBC (10^6mm^3^)	4.54	4.07-5.86	4.45	3.83-5.26	4.36	3.23-5.21	0.0811
Hemoglobin (g/dl)	11.50	10.19-13.90	11.90	10.2-13.5	11.35	8.61-13.20	0.0067
Hematocrit (%)	37.95	34.05-45.04	35.90	30.7-40.4	34.40	27.01-40.59	0.0654
MCV (*μ*mm^3^)	85.00	71.90-95.03	79.00	64-92	81.00	70.00-88.97	0.0125
MCH (pg)	25.50	21.25-36.06	26.60	20.8-30.9	26.45	22.20-30.28	≤0.001
MCHC (g/dl)	30.60	28.80-303.07	33.50	32.3-35.2	32.80	31.4-34.3	0.9092
Platelet (1000/mm^3^)	263.00	189.83-389.52	276.00	22-386	271.50	83.60-599.23	0.0196
MPV (*μ*m^3^)	8.25	7.29-9.90	8.40	7.0-10.2	7.90	6.4-9.2	0.2899
Lymphocytes (%)	47.60	29.43-76.63	46.50	30.1-58.0	48.10	26.46-59.95	0.0011
Monocytes (%)	10.65	7.08-16.81	9.40	6.5-12.4	10.45	5.81-16.09	0.1637
Neutrophils (%)	32.55	20.68-54.23	38.40	23.1-56.7	36.85	21.01-62.44	0.3907
Eosinophils (%)	4.05	1.10-16.45	3.20	1.2-21.5	3.20	0.91-21.47	0.1948
Basophils (%)	0.60	0.4-1.0	0.70	0.3-1.0	0.60	0.3-0.9	≤0.001
AST (UI/L)	30.00	22.97-34.02	24.30	12.3-38.4	17.10	9.82-27.20	≤0.001
ALT (UI/L)	20.00	12.95-28.05	13.60	9.10-27.55	9.10	9.10-14.57	≤0.001
T/Bil (UI/L)	0.65	0.50-1.12	0.40	0.2-0.9	0.30	0.2-0.9	≤0.001
Creatinine (mg/L)	0.60	0.4-0.8	0.64	0.2-0.9	0.66	0.18-0.86	0.5514

∗*p* value based on Friedman test.

**Table 6:** Reference values among Female cohort for 16-45 years of age who attended all rounds (1 to 3). *N* = 32.

Parameters (unit)	1st round (dry season)		2nd round (rainy season)		3rd round (end of rainy season)		∗*p* value
	Median	References value	Median	References value	Median	References value	
WBC (1000/mm^3^)	6.40	4.03-8.85	6.90	4.45-11.80	6.30	4.21-8.78	0.1615
RBC (10^6mm^3^)	4.46	3.62-5.71	4.41	3.67-5.69	4.38	3.41-5.27	0.773
Hemoglobin (g/dl)	11.40	7.82-13.62	11.90	9.22-14.00	11.70	8.40-13.3S6	0.1679
Hematocrit (%)	37.60	26.87-44.17	35.50	28.85-41.73	35.85	27.68-40.46	0.0829
MCV (*μ*mm^3^)	83.50	62.95-95.45	81.00	63.75-93.75	80.00	66.90-94.10	0.5783
MCH (pg)	25.35	16.48-82.35	27.30	20.30-32.65	26.45	20.23-30.95	0.0976
MCHC(g/dl)	30.35	28.18-31.69	33.10	28.00-35.55	32.70	30.12-33.80	≤0.001
Platelet (1000/mm^3^)	236.00	119.65-407.70	258.00	138-406	265.00	125.60-447.07	0.1203
MPV (*μ*m^3^)	8.35	7.46-9.55	8.40	7.05-10.07	8.60	7.04-10.33	0.3585
Lymphocytes (%)	41.60	31.19-63.55	41.30	28.43-60.50	44.70	26.39-58.72	0.3607
Monocytes (%)	9.85	5.38-14.73	9.20	5.73-15.73	8.90	5.37-14.69	0.6763
Neutrophils (%)	38.45	21.28-54.22	41.90	22.62-58.62	38.65	24.53-61.95	0.327
Eosinophils (%)	5.85	1.41-20.54	3.30	0.82-17.62	2.90	0.87-21.20	0.0528
Basophils (%)	0.60	0.38-7.22	0.60	0.32-1.05	0.60	0.40-0.98	0.4169
AST (UI/L)	28.50	22.33-40.67	26.40	15.05-43.30	19.40	9.20-30.99	≤0.001
ALT (UI/L)	19.00	13.77-50.60	14.35	9.10-37.31	10.05	9.1-27.3	≤0.001
T/Bil (UI/L)	0.60	0.40-0.82	0.50	0.40-0.82	0.30	0.20-1.11	≤0.001
Creatinine (mg/L)	0.70	0.30-0.92	0.73	0.30-0.92	0.78	0.36-1.02	0.0992

^∗^ *p* value based on Friedman test.

**Table 7:** Reference values among Male cohort 6-10 years of age who attended all rounds (1 to 3). *N* = 46.

Parameters (unit)	1st round (dry season)		2nd round (rainy season)		3rd round (end of rainy season)		∗*p* value
	Median	References value	Median	References value	Median	References value	
WBC (1000/mm^3^)	8.10	4.50-16.23	7.60	4.55-12.80	7.60	4.85-12.32	0.2596
RBC (10^6mm^3^)	4.56	3.44-5.71	4.50	3.88-5.37	4.33	3.25-5.20	0.7266
Hemoglobin (g/dl)	11.20	7.83-12.19	11.10	9.6-12.7	11.10	8.04-13.15	0.0096
Hematocrit (%)	36.80	26.62-40.34	33.10	28.6-37.7	33.90	25.65-39.35	≤0.001
MCV (*μ*mm^3^)	78.00	15.88-88.00	74.00	59.5-85.5	79.00	67-85	0.0046
MCH (pg)	23.75	19.81-27.34	24.80	19.55-28.70	25.80	21.23-28.21	0.0022
MCHC (g/dl)	30.40	28.71-31.95	33.40	31.5-35.1	32.70	30.50-34.77	≤0.001
Platelet (1000/mm^3^)	305.00	60.88-445.62	288.00	88.2-467.5	304.00	151.25-476.75	0.3735
MPV (*μ*m^3^)	8.20	6.91-9.29	8.20	6.80-9.80	7.90	6.80-9.70	0.8298
Lymphocytes (%)	47.60	28.94-61.96	46.40	27.25-60.15	43.70	31.06-62.38	0.4553
Monocytes (%)	10.45	6.83-15.64	9.80	6.95-15.30	10.50	6.48-19.09	0.2364
Neutrophils (%)	34.10	21.66-53.11	35.90	22.20-58.95	36.30	21.67-67.38	0.05803
Eosinophils (%)	4.40	0.85-20.76	4.70	1.2-20.7	4.20	1.03-26.76	0.5881
Basophils (%)	0.75	0.41-3.64	0.60	0.35-0.90	0.70	0.45-1.05	≤0.001
AST (UI/L)	37.00	29.00-55.75	31.70	21.15-57.45	23.10	13.08-57.24	≤0.001
ALT (UI/L)	22.00	16.12-50.25	20.90	10.81-59.84	10.65	9.10-35.85	≤0.001
T/Bil (UI/L)	0.60	0.40-2.54	0.30	0.20-0.85	0.40	0.20-1.17	≤0.001
Creatinine (mg/L)	0.50	0.3-0.7	0.58	0.40-0.69	0.56	0.39-0.77	≤0.001

^∗^ *p* value based on Friedman test.

**Table 8:** Reference values among Male cohort 11-15 years of age who attended all rounds (1 to 3). *N* = 32.

Parameters (unit)	1^st^ round (dry season)		2^nd^ round (rainy season)		3^rd^ round (end of rainy season)		∗*p* value
	Median	References value	Median	References value	Median	References value	
WBC (1000/mm^3^)	6.55	4.28-10.69	6.50	3.96-10.25	5.90	4.90-7.76	0.3563
RBC (10^6mm^3^)	4.59	4.14-5.61	4.50	3.92-5.28	4.46	3.16-5.46	0.1184
Hemoglobin (g/dl)	11.65	9.91-13.60	11.80	9.96-12.94	11.30	7.32-14.18	0.0011
Hematocrit (%)	38.10	32.60-44.46	35.60	30.49-37.45	34.40	24.64-42.28	0.0617
MCV (*μ*mm^3^)	82.00	71.88-87.00	79.00	66.5-86.0	78.00	62.6-87.8	0.0946
MCH (pg)	25.00	20.90-26.95	26.20	20.98-29.05	25.80	19.04-29.14	0.0074
MCHC (g/dl)	30.60	29.46-31.62	33.70	31.45-34.99	32.60	30.12-34.76	≤0.001
Platelet (1000/mm^3^)	252.50	180.40-389.57	267.50	121.00-347.75	278.00	179.0-347.4	0.5523
MPV (*μ*m^3^)	8.20	7.15-9.58	8.15	7.01-9.56	8.20	6.88-9.42	0.9447
Lymphocytes (%)	44.60	30.98-51.37	48.45	33.86-59.48	47.30	32.10-58.16	0.1301
Monocytes (%)	10.20	6.27-21.07	9.75	5.21-13.93	10.80	7.74-18.24	0.1919
Neutrophils (%)	35.75	26.27-51.35	33.10	22.05-50.11	35.90	20.24-51.30	0.348
Eosinophils (%)	6.30	1.54-15.99	6.60	2.21-27.10	6.10	1.28-13.62	0.4444
Basophils (%)	0.70	0.38-2.03	0.60	0.36-0.84	0.60	0.40-1.18	0.0761
AST (UI/L)	34.50	26.32-53.80	29.00	15.03-45.15	22.40	12.92-34.68	≤0.001
ALT (UI/L)	22.50	15.32-41.12	19.14	9.88-32.65	12.02	9.10-20.25	≤0.001
T/Bil (mg/L)	0.60	0.48-0.82	0.30	0.20-0.94	0.30	0.20-5.42	≤0.001
Creatinine (mg/L)	0.70	0.48-0.90	0.68	0.24-0.92	0.74	0.50-0.97	0.6065

^∗^ *p* value based on Friedman test.

**Table 9:** Reference values for Male cohort 16-45 years of age who attended all rounds (1 to 3). *N* = 32.

Parameters (unit)	1^st^ round (dry season)		2^nd^ round (rainy season)			3^rd^ round (end of rainy season)		*p* value
	Median	References value	Median	References value	N	Median	References value	
WBC (1000/mm^3^)	6.40	4.11-11.04	6.25	3.14-9.97		6.30	4.21-8.78	0.346
RBC (10^6mm^3^)	5.11	4.37-6.08	4.98	4.17-6.26		4.38	4.21-8.78	0.0651
Hemoglobin (g/dl)	13.40	10.41-14.85	13.85	10.62-15.50		11.70	3.41-5.27	0.755
Hematocrit (%)	44.60	34.95-49.45	40.80	31.02-46.14		35.85	8.40-13.36	≤0.001
MCV (*μ*mm^3^)	84.00	68.60-95.00	83.00	67.25-91.88		80.00	66.9-94.1	0.3171
MCH (pg)	25.80	20.32-29.23	27.15	21.31-31.14		26.45	20.23-30.95	0.0015
MCHC (g/dl)	30.50	29.24-31.06	33.20	31.95-34.84		32.70	30.12-33.80	≤0.001
Platelet (1000/mm^3^)	222.00	101.50-414.80	209.00	50.00-735.12		265.00	125.60-447.07	0.9382
MPV (*μ*m^3^)	8.70	7.28-10.03	8.50	6.81-9.59		8.60	7.04-10.33	0.0207
Lymphocytes (%)	44.70	26.68-65.61	47.05	27.18-61.14		44.70	26.39-58.72	0.3526
Monocytes (%)	9.90	7.34-16.05	9.25	6.54-13.46		8.90	5.37-14.69	0.0116
Neutrophils (%)	33.60	19.56-62.17	36.15	20.86-61.11		38.65	24.53-61.95	0.0274
Eosinophils (%)	5.40	1.17-20.73	6.05	1.21-18.86		2.90	0.87-21.20	0.2758
Basophils (%)	0.70	0.40-2.73	0.60	0.31-1.08		0.60	0.40-0.98	0.0668
AST (UI/L)	33.00	24.4-76.0	26.65	14.97-43.75		19.40	9.20-30.99	≤0.001
ALT (UI/L)	20.00	14.7-43.3	19.11	9.34-42.13		10.05	9.1-27.3	≤0.001
T/Bil (UI/L)	0.70	0.50-0.96	0.60	0.21-3.83		0.30	0.20-1.11	≤0.001
Creatinine (mg/L)	1.00	0.27-1.33	0.96	0.39-1.21		0.78	0.36-1.02	0.0715

^∗^ *p* value based on Friedman test.

## Data Availability

The datasets in csv Excel format and the software R scripts used to support the findings of this study are available from the corresponding author upon request.

## Supplementary Materials

Supplementary Materials The supplementary material includes the Pairwise Analysis
