# Supplementary material for: Haematological and Biochemical Reference Values for Healthy Population of Maferinyah Rural Community in Guinea
Source: Biomed Res Int. 2020 Aug 3;2020:8605485. doi: 10.1155/2020/8605485 (PMC7421051; doi:10.1155/2020/8605485)
Supplement: Supplementary Materials — The supplementary material includes the Pairwise Analysis [file 8605485.f1.pdf]

Table4: Pairwise analysis

```
> posthoc.friedman.nemenyi.test(P13)

        Pairwise comparisons using Nemenyi multiple comparison test
             with q approximation for unreplicated blocked data

data:  P13

      HTF11   HTF21
HTF21 0.00244 -
HTF31 0.00071 0.94046

P value adjustment method: none


> posthoc.friedman.nemenyi.test(P14)

        Pairwise comparisons using Nemenyi multiple comparison test
             with q approximation for unreplicated blocked data

data:  P14

       VGMF11 VGMF21
VGMF21 0.051  -
VGMF31 0.124  0.923

P value adjustment method: none
> posthoc.friedman.nemenyi.test(P104)

        Pairwise comparisons using Nemenyi multiple comparison test
             with q approximation for unreplicated blocked data

data:  P104

        ASATF11 ASATF21
ASATF21 0.0024  -
ASATF31 2.8e-11 0.0015

P value adjustment method: none
> posthoc.friedman.nemenyi.test(P105)

        Pairwise comparisons using Nemenyi multiple comparison test
             with q approximation for unreplicated blocked data
```

```
data:  P105

       ALTF11  ALTF21
ALTF21 0.67    -
ALTF31 1.4e-10 3.2e-08

P value adjustment method: none
> posthoc.friedman.nemenyi.test(P106)

        Pairwise comparisons using Nemenyi multiple comparison test
             with q approximation for unreplicated blocked data

data:  P106

              TBILIRIBUNF11 TBILIRIBUNF21
TBILIRIBUNF21 0.0047        -
TBILIRIBUNF31 3.5e-06       0.1991

P value adjustment method: none
> posthoc.friedman.nemenyi.test(P107)

        Pairwise comparisons using Nemenyi multiple comparison test
             with q approximation for unreplicated blocked data

data:  P107

              CREATININEF11 CREATININEF21
CREATININEF21 0.00012       -
CREATININEF31 0.00027       0.98015

P value adjustment method: none
```

Table 5: Pairwise analysis

```
> posthoc.friedman.nemenyi.test(p161)

        Pairwise comparisons using Nemenyi multiple comparison test
             with q approximation for unreplicated blocked data

data:  p161

      GBF12  GBF22
GBF22 0.0324 -
GBF32 0.6574 0.0021
```

```
P value adjustment method: none
> posthoc.friedman.nemenyi.test(P132)

	Pairwise comparisons using Nemenyi multiple comparison test	
             with q approximation for unreplicated blocked data 

data:  P132 

      HTF12 HTF22
HTF22 0.037 -    
HTF32 0.009 0.876

P value adjustment method: none 


> posthoc.friedman.nemenyi.test(P151)

	Pairwise comparisons using Nemenyi multiple comparison test	
             with q approximation for unreplicated blocked data 

data:  P151 

        TGMHF12 TGMHF22
TGMHF22 0.021   -      
TGMHF32 0.043   0.960  

P value adjustment method: none 


> posthoc.friedman.nemenyi.test(P152)

	Pairwise comparisons using Nemenyi multiple comparison test	
             with q approximation for unreplicated blocked data 

data:  P152 

        CCMHF12 CCMHF22
CCMHF22 1.7e-13 -      
CCMHF32 1.4e-05 0.009  

P value adjustment method: none 
```

```
> posthoc.friedman.nemenyi.test(P171)

        Pairwise comparisons using Nemenyi multiple comparison test
              with q approximation for unreplicated blocked data

data:  P171

       VMPF12 VMPF22
VMPF22 0.554  -
VMPF32 0.018  0.209

P value adjustment method: none

> posthoc.friedman.nemenyi.test(P191)

        Pairwise comparisons using Nemenyi multiple comparison test
              with q approximation for unreplicated blocked data

data:  P191

       MONF12  MONF22
MONF22 0.00093 -
MONF32 0.38993 0.05701

P value adjustment method: none
> posthoc.friedman.nemenyi.test(P1032)

        Pairwise comparisons using Nemenyi multiple comparison test
              with q approximation for unreplicated blocked data

data:  P1032

        ASATF12 ASATF22
ASATF22 0.024   -
ASATF32 1.9e-11 6.2e-05

P value adjustment method: none
> posthoc.friedman.nemenyi.test(P1033)

        Pairwise comparisons using Nemenyi multiple comparison test
              with q approximation for unreplicated blocked data

data:  P1033

       ALTF12  ALTF22
ALTF22 0.032   -
```

```
ALTF32 8.2e-13 4.7e-06

P value adjustment method: none
> posthoc.friedman.nemenyi.test(P1041)

        Pairwise comparisons using Nemenyi multiple comparison test
             with q approximation for unreplicated blocked data

data:  P1041

              TBILIRIBUNF12 TBILIRIBUNF22
TBILIRIBUNF22 0.0014        -
TBILIRIBUNF32 8.1e-06       0.4530

P value adjustment method: none
```

Table 6: Pairwise analysis

```
> posthoc.friedman.nemenyi.test(P352)

        Pairwise comparisons using Nemenyi multiple comparison test
             with q approximation for unreplicated blocked data

data:  P352

        CCMHF13 CCMHF23
CCMHF23 9.2e-14 -
CCMHF33 1.5e-07 0.075

P value adjustment method: none
> posthoc.friedman.nemenyi.test(P3033)

        Pairwise comparisons using Nemenyi multiple comparison test
             with q approximation for unreplicated blocked data

data:  P3033

        ALTF13 ASATF23
ASATF23 0.046  -
ASATF33 0.953  0.021

P value adjustment method: none
> posthoc.friedman.nemenyi.test(P3041)
```

```
        Pairwise comparisons using Nemenyi multiple comparison test
             with q approximation for unreplicated blocked data

data:  P3041

              TBILIRIBUNF13 TBILIRIBUNF23
TBILIRIBUNF23 0.0037        -
TBILIRIBUNF33 4.6e-07       0.1051

P value adjustment method: none
```

Table 7:

```
> posthoc.friedman.nemenyi.test(p422)

        Pairwise comparisons using Nemenyi multiple comparison test
             with q approximation for unreplicated blocked data

data:  p422

      GRM11  GRF21
GRF21 0.5689 -
GRM31 0.0078 0.1173

P value adjustment method: none
> posthoc.friedman.nemenyi.test(P432)

        Pairwise comparisons using Nemenyi multiple comparison test
             with q approximation for unreplicated blocked data

data:  P432

      HTM11   HTM21
HTM21 4.1e-05 -
HTM31 0.0012  0.6938

P value adjustment method: none
> posthoc.friedman.nemenyi.test(P441)
```

```
        Pairwise comparisons using Nemenyi multiple comparison test
             with q approximation for unreplicated blocked data

data:  P441

       VGMM11 VGMM21
VGMM21 0.0068 -
VGMM31 0.8015 0.0434

P value adjustment method: none
> posthoc.friedman.nemenyi.test(P451)

        Pairwise comparisons using Nemenyi multiple comparison test
             with q approximation for unreplicated blocked data

data:  P451

        TGMHM11 TGMHM21
TGMHM21 0.3160  -
TGMHM31 0.0017  0.1142

P value adjustment method: none
> posthoc.friedman.nemenyi.test(P452)

        Pairwise comparisons using Nemenyi multiple comparison test
             with q approximation for unreplicated blocked data

data:  P452

        CCMHM11 CCMHM21
CCMHM21 2.6e-14 -
CCMHM31 7.0e-10 0.061

P value adjustment method: none
> posthoc.friedman.nemenyi.test(P4001)

        Pairwise comparisons using Nemenyi multiple comparison test
             with q approximation for unreplicated blocked data

data:  P4001

       NEUM11 NEUM21
NEUM21 0.551  -
NEUM31 0.034  0.316

P value adjustment method: none
```

```
> posthoc.friedman.nemenyi.test(P4022)

        Pairwise comparisons using Nemenyi multiple comparison test
             with q approximation for unreplicated blocked data

data:  P4022

       BASM11  BASM21
BASM21 6.2e-05 -
BASM31 0.0091  0.3880

P value adjustment method: none
> posthoc.friedman.nemenyi.test(P4032)

        Pairwise comparisons using Nemenyi multiple comparison test
             with q approximation for unreplicated blocked data

data:  P4032

        ASATM11 ASATM21
ASATM21 0.024   -
ASATM31 3.6e-13 4.8e-06

P value adjustment method: none
> posthoc.friedman.nemenyi.test(P4033)

        Pairwise comparisons using Nemenyi multiple comparison test
             with q approximation for unreplicated blocked data

data:  P4033

       ALTM11  ALTM21
ALTM21 0.47    -
ALTM31 9.4e-12 2.1e-08

P value adjustment method: none
> posthoc.friedman.nemenyi.test(P4041)

        Pairwise comparisons using Nemenyi multiple comparison test
             with q approximation for unreplicated blocked data

data:  P4041

             TBILIRIBUNM11 TBILIRIBUNM21
TBILIRIBUNM21 1.9e-06       -
TBILIRIBUNM31 6.2e-05       0.75
```

```
P value adjustment method: none
> posthoc.friedman.nemenyi.test(P4051)

	Pairwise comparisons using Nemenyi multiple comparison test	
             with q approximation for unreplicated blocked data 

data:  P4051 

              CREATININEM11 CREATININEM21
CREATININEM21 0.00042       -            
CREATININEM31 0.00320       0.84994      

P value adjustment method: none
```

Table 8 : Pairwise analysis

```
> posthoc.friedman.nemenyi.test(P532)

	Pairwise comparisons using Nemenyi multiple comparison test	
             with q approximation for unreplicated blocked data 

data:  P532 

      HTM12  HTM22 
HTM22 0.0041 -     
HTM32 0.0041 1.0000

P value adjustment method: none
> posthoc.friedman.nemenyi.test(P551)

	Pairwise comparisons using Nemenyi multiple comparison test	
             with q approximation for unreplicated blocked data 

data:  P551 

        TGMHM12 TGMHM22
TGMHM22 0.0051  -      
TGMHM32 0.3541  0.1868 
```

```
P value adjustment method: none
> posthoc.friedman.nemenyi.test(P552)

	Pairwise comparisons using Nemenyi multiple comparison test
	     with q approximation for unreplicated blocked data

data:  P552

        CCMHM12 CCMHM22
CCMHM22 6.8e-11 -
CCMHM32 1.5e-05 0.085

P value adjustment method: none
> posthoc.friedman.nemenyi.test(P5032)

	Pairwise comparisons using Nemenyi multiple comparison test
	     with q approximation for unreplicated blocked data

data:  P5032

        ASATM12 ASATM22
ASATM22 0.0393  -
ASATM32 1.6e-07 0.0076

P value adjustment method: none
> posthoc.friedman.nemenyi.test(P5033)

	Pairwise comparisons using Nemenyi multiple comparison test
	     with q approximation for unreplicated blocked data

data:  P5033

       ALTM12  ALTM22
ALTM22 0.08482 -
ALTM32 2.7e-09 0.00019

P value adjustment method: none
> posthoc.friedman.nemenyi.test(P5041)

	Pairwise comparisons using Nemenyi multiple comparison test
	     with q approximation for unreplicated blocked data

data:  P5041

              TBILIRIBUNM12 TBILIRIBUNM22
```

```
TBILIRIBUNM22 8.4e-05       -
TBILIRIBUNM32 0.00066       0.87131

P value adjustment method: none
```

Table 9 : Pairwise Analysis

```
> posthoc.friedman.nemenyi.test(P632)

        Pairwise comparisons using Nemenyi multiple comparison test
             with q approximation for unreplicated blocked data

data:  P632

      HTM13  HTM23
HTM23 0.0028 -
HTM33 0.0040 0.9942

P value adjustment method: none
> posthoc.friedman.nemenyi.test(P651)

        Pairwise comparisons using Nemenyi multiple comparison test
             with q approximation for unreplicated blocked data

data:  P651

        TGMHM13 TGMHM23
TGMHM23 0.0047  -
TGMHM33 0.0066  0.9942

P value adjustment method: none
> posthoc.friedman.nemenyi.test(P652)

        Pairwise comparisons using Nemenyi multiple comparison test
             with q approximation for unreplicated blocked data

data:  P652

        CCMHM13 CCMHM23
CCMHM23 5.9e-14 -
CCMHM33 4.8e-10 0.32

P value adjustment method: none
> posthoc.friedman.nemenyi.test(P671)
```

```
        Pairwise comparisons using Nemenyi multiple comparison test
             with q approximation for unreplicated blocked data

data:  P671

       VMPM13 VMPM23
VMPM23 0.590  -
VMPM33 0.017  0.186

P value adjustment method: none
> posthoc.friedman.nemenyi.test(P691)

        Pairwise comparisons using Nemenyi multiple comparison test
             with q approximation for unreplicated blocked data

data:  P691

       MONM13 MONM23
MONM23 0.046  -
MONM33 0.931  0.017

P value adjustment method: none
> posthoc.friedman.nemenyi.test(P6001)

        Pairwise comparisons using Nemenyi multiple comparison test
             with q approximation for unreplicated blocked data

data:  P6001

       NEUM13 NEUM23
NEUM23 0.37   -
NEUM33 0.02   0.37

P value adjustment method: none
> posthoc.friedman.nemenyi.test(P6032)

        Pairwise comparisons using Nemenyi multiple comparison test
             with q approximation for unreplicated blocked data

data:  P6032

        ASATM13 ASATM23
ASATM23 0.00011 -
ASATM33 1.7e-10 0.04069
```

```
P value adjustment method: none
> posthoc.friedman.nemenyi.test(P6033)

        Pairwise comparisons using Nemenyi multiple comparison test
             with q approximation for unreplicated blocked data

data:  P6033

       ALTM13  ALTM23
ALTM23 0.97681 -
ALTM33 0.00011 0.00026

P value adjustment method: none
> posthoc.friedman.nemenyi.test(P6041)

        Pairwise comparisons using Nemenyi multiple comparison test
             with q approximation for unreplicated blocked data

data:  P6041

              TBILIRIBUNM13 TBILIRIBUNM23
TBILIRIBUNM23 0.0977        -
TBILIRIBUNM33 2.4e-07       0.0028

P value adjustment method: none
```
